# Predictive model for microclimatic temperature and its use in mosquito population modeling

**DOI:** 10.1038/s41598-021-98316-x

**Published:** 2021-09-23

**Authors:** Madhav Erraguntla, Darpit Dave, Josef Zapletal, Kevin Myles, Zach N. Adelman, Tyler D. Pohlenz, Mark Lawley

**Affiliations:** 1 Department of Industrial Engineering, Texas A&M University, College Station, USA; 2 Department of Entomology, Texas A&M University, College Station, USA

**Keywords:** Infectious diseases, Scientific data, Computational models, Data mining, Machine learning

## Abstract

Mosquitoes transmit several infectious diseases that pose significant threat to human health. Temperature along with other environmental factors at breeding and resting locations play a role in the organismal development and abundance of mosquitoes. Accurate analysis of mosquito population dynamics requires information on microclimatic conditions at breeding and resting locations. In this study, we develop a regression model to characterize microclimatic temperature based on ambient environmental conditions. Data were collected by placing sensor loggers at resting and breeding locations such as storm drains across Houston, TX. Corresponding weather data was obtained from National Oceanic and Atmospheric Administration website. Features extracted from these data sources along with contextual information on location were used to develop a Generalized Linear Model for predicting microclimate temperatures. We also analyzed mosquito population dynamics for *Aedes albopictus* under ambient and microclimatic conditions using system dynamic (SD) modelling to demonstrate the need for accurate microclimatic temperatures in population models. The microclimate prediction model had an R^2^ value of ~ 95% and average prediction error of ~ 1.5 °C indicating that microclimate temperatures can be reliably estimated from the ambient environmental conditions. SD model analysis indicates that some microclimates in Texas could result in larger populations of juvenile and adult *Aedes albopictus* mosquitoes surviving the winter without requiring dormancy.

## Introduction

Vector-borne diseases have become a major public health crisis globally. In addition to high morbidity and mortality rates from malaria and dengue fever, recent epidemics of Zika, West Nile and chikungunya have put nearly 4 billion people at risk across countries around the world^1–4^. Due to the role mosquitoes serve in the transmission of these diseases and nuisance created by the mosquito bites, it is critical to study the factors that influence growth and abundance of mosquito populations^5,6^.

There is considerable literature available to support that environmental conditions including temperature affect growth of mosquito populations as well as their ability to transmit diseases^4,7–19^. Mosquitos, in part due to their physiological structure and characteristics, display a complex and nonlinear epidemiological relationship to temperature^20–22^. Many works often make unrealistic assumptions about the existence of a simplistic relationship between the mosquito population and the environment^23–26^. In addition, the effects of temperature on mosquitoes vary based on different life stages^27–29^. Population growth parameters in different stages of the life cycle such as development rate, fecundity, reproduction rate, and survival rates need to be modelled as temperature dependent for accurate population modelling^4,30–36^.

Previous works have mostly relied on ambient environmental conditions to develop mosquito population models^29,37–42^. These environmental conditions are derived from weather stations or remotely-sensed datasets and used for predicting mosquito presence, population growth rates, and transmission dynamics. Some works have tried to include the landscape data in addition to the ambient climatic data^12,43^. Ambient conditions could differ significantly from microclimatic conditions in some breeding and resting locations^44,45^. Some microclimatic locations support for higher temperatures in the winter and night times compared to ambient conditions potentially benefiting mosquito population dynamics. As a result, development of population models based on microclimatic conditions, rather than ambient conditions, will facilitate more accurate population modelling and analysis.

Microclimatic conditions and their effect on mosquito population have been studied before^44,45^. These approaches need microclimatic data and necessitate placement of a sensor suite to collect, aggregate, process, and analyze microclimatic conditions, thereby making it expensive and impractical for large scale deployment and wide adoption. In this work, we develop a regression model to characterize microclimatic temperature in storm drains, a mosquito breeding and resting site, as a function of ambient environmental conditions. Such a model will facilitate accurate estimation of microclimatic temperature from ambient conditions, without necessitating deployment of an elaborate sensor-based data collection system. Extension of the predictive model developed to other microclimatic conditions (humidity, light intensity lux) and sub-tropical conditions will help account for multivariate effects of such factors, result in more flexible and generalizable models and will alleviate the need for microclimatic data collection in such locations.

## Materials and methods

### Data collection

**Figure 1:**
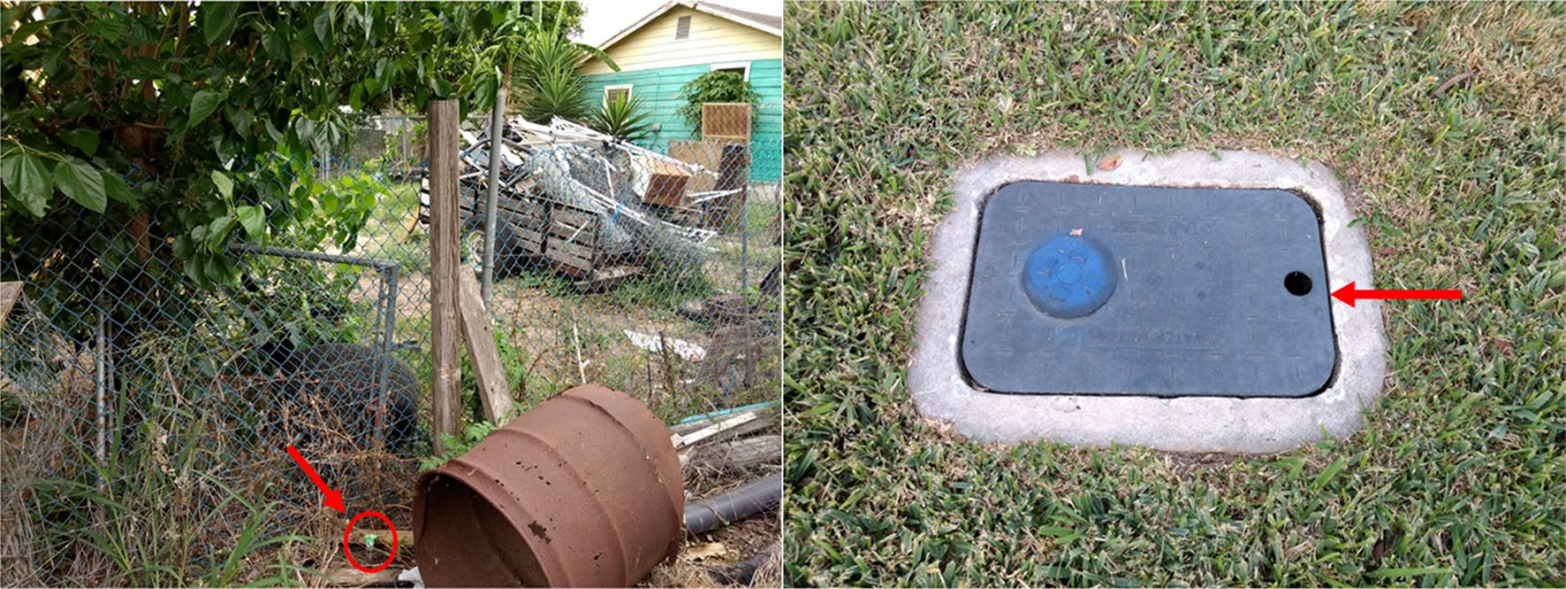
Logger placements: (left) in the open, (right) inside water meter.

**Figure 2:**
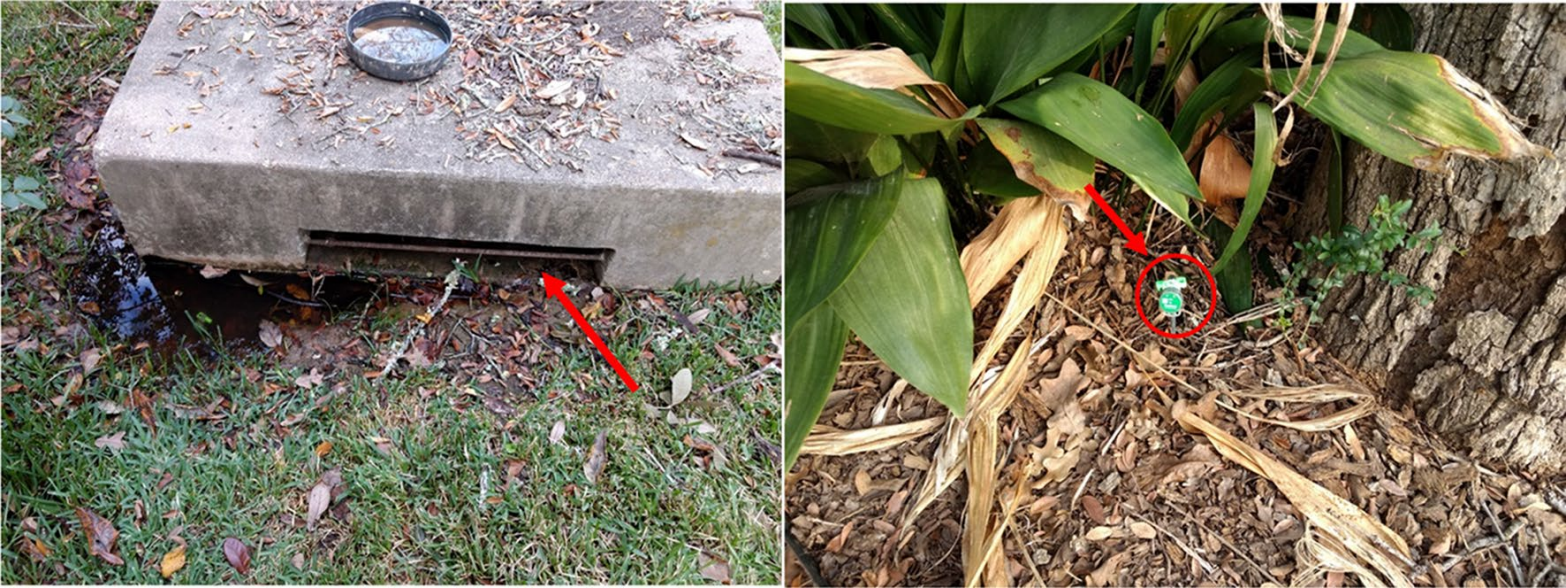
Logger placements: (left) inside storm drain, (right) under the shade.

Microclimatic data was collected by placing data loggers (Onset Computer Corporation, Bourne, MA, USA: HOBO Pendant MX Temperature/Light Data Logger, MX2202) at active mosquito trap sites across Harris County, Texas. In addition, to capture the microclimate heterogeneity in the natural urban habitats of mosquitoes, data loggers were deployed in exposed and shaded sites, as well as subterranean locations such as storm drains and water meter boxes (Figs. 1, 2). A total of thirty-five data loggers were deployed at different sites, with twenty-five loggers being placed in storm drains and ten being placed near mosquito traps at residences, however due to damage and theft, only a subset of the data loggers (8) placed in storm drains provided useable data for the entire study period (May 2018 to June 2019). Instantaneous temperatures were recorded at ten-minute intervals throughout the study period.

Hourly ambient climate data was obtained from the NOAA repository^46^. Data logger locations were mapped to the nearest zip code and through zip code to ambient NOAA data. This facilitated mapping of microclimatic temperature to ambient environmental conditions.

### Methodology

#### Exploratory data analysis

**Figure 3:**
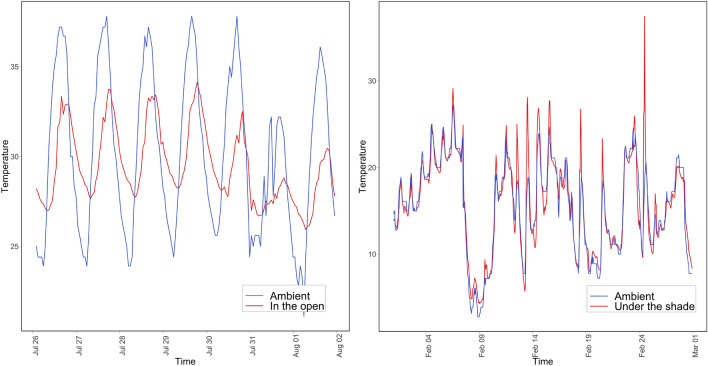
Ambient versus microclimatic temperatures (left) in open (right) tree coverage.

**Figure 4:**
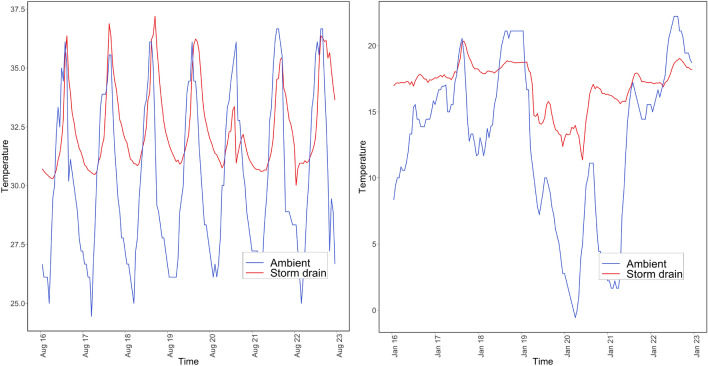
Ambient versus microclimatic temperatures in storm drain location (L) in August (R) in January.

**Figure 5:**
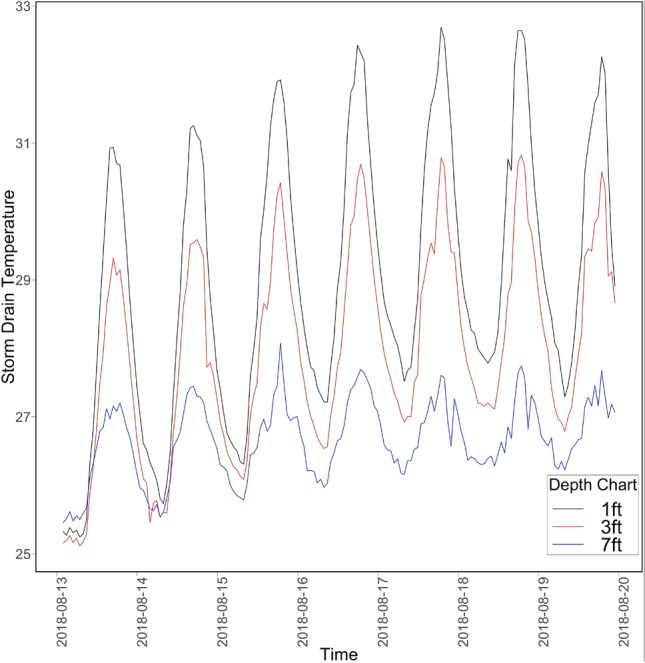
Storm drain temperature recorded at different depths.

As part of exploratory data analysis, microclimatic and ambient temperature patterns at different sensor locations were analyzed. Microclimatic temperature patterns are closely correlated to ambient patterns for exposed and shaded locations (Fig. 3 shows sample pattern from one location). However, the microclimatic air temperature differs significantly from ambient conditions for storm drains (Fig. 4 shows sample pattern from one location). It appears that storm drains provide a thermal insulation effect and facilitate warmer temperatures within these locations during the colder ambient temperatures. Depth of the storm drain was another factor influencing the difference in temperature patterns (Fig. 5 shows sample pattern at three depth locations). Based on these observations, the microclimatic model prediction was focused on storm drains at three different depths of 1, 3, and 7 ft in this study and depth was included as a predictor variable.

**Figure 6:**
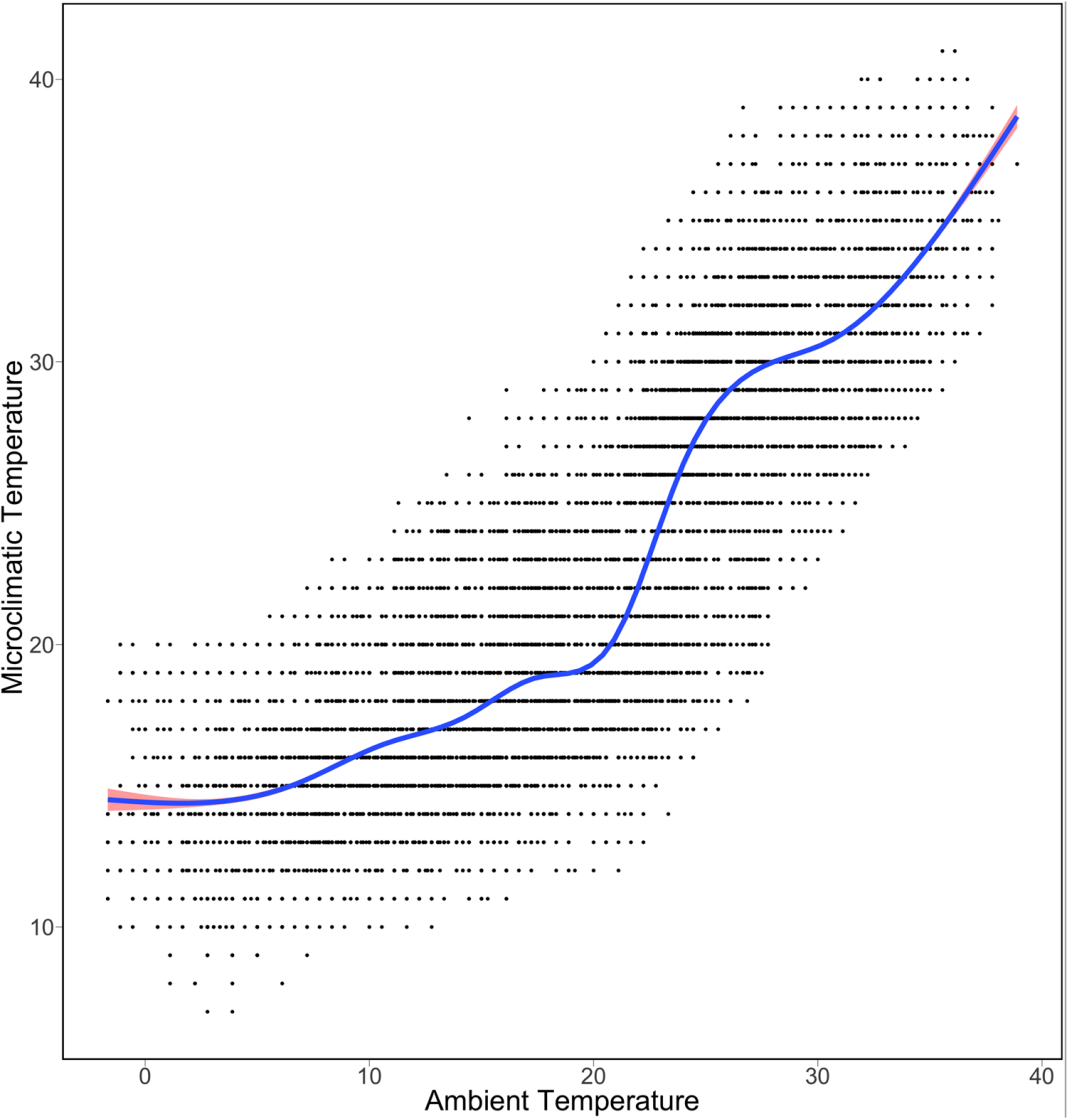
Scatter plot indicating non-linear relationship between ambient and microclimatic temperatures.

It also appears that there is a non-linear relationship between microclimatic temperature and ambient temperature (Fig. 6), indicating the need for non-linear features in the predictive model and the potential influence of other predictor variables. Figure 6 shows a scatter plot between microclimatic and ambient temperature based on 8 storm drain logger data. The curve fitting was performed using a generalized additive model.

Microclimate prediction model.

A linear regression model (with non-linear features) was developed to predict microclimatic temperature in the storm drains using ambient weather conditions. Weather related ambient features extracted from NOAA data included ambient temperature, precipitation, relative humidity, lux and air pressure. A rich set of non-linear and linear features extracted from ambient weather, as well as contextual information (hour of the day, month, and logger depth) were used as predictor variables. Diurnal temperature range defined as the range between maximum and minimum ambient temperature in the last 24 h was included as a predictor variable. Moving average and time-lagged features were included to capture the temporal effects. The microclimate temperature was used as the response variable. The list of features used in the model are presented in Table 1.

**Table 1:** Variables used for modelling.

Variable name	Description	Estimate	*P* value
*A*			
**Ambient climate features**			
Lux	Luminous intensity	− 0.00016	0.00222
Hourly Dew Point Temperature	Dew point temperature	− 0.05691	< 0.00001
Hourly Dry Bulb Temperature	Ambient temperature	− 0.1493	< 0.00001
Hourly Precipitation	Amount of rainfall recorded every hour	− 0.90674	< 0.00001
Hourly Relative Humidity	Relative humidity	0.01502	< 0.00001
Hourly Station Pressure	Pressure obtained from the weather sensor	− 1.41615	< 0.00001
Hourly Wet Bulb Temperature	Wet bulb temperature	− 0.05448	0.00001
Diurnal Range	Difference between the maximum and minimum ambient temperature recorded in the past 24 Hours	0.10055	< 0.00001
3 Hour Moving Average	Moving average of ambient temperature in the past 3 h	0.09778	< 0.00001
5 Hour Moving Average	Moving average of ambient temperature in the past 5 h	− 0.17148	< 0.00001
7 Hour Moving Average	Moving average of Dry bulb temperature in the past 7 h	0.27072	< 0.00001
Square of Diurnal Range Square		− 0.00293	< 0.00001
Square of Ambient Temperature		0.00192	< 0.00001
*B*			
**Contextual variables**			
Depth	Depth of the logger	− 0.32745	< 0.00001
Month (Jan Baseline)	Month of the year		
Month 2		0.64201	< 0.00001
Month 3		1.57424	< 0.00001
Month 6		8.17883	< 0.00001
Month 7		9.80838	< 0.00001
Month 8		9.58742	< 0.00001
Month 9		8.33488	< 0.00001
Month 10		6.6148	< 0.00001
Month 11		3.55702	< 0.00001
Month 12		1.16736	< 0.00001
Time (Hour 24 Baseline)	Hour of the day		< 0.00001
Hour 1		0.11006	0.04926
Hour 2		0.11144	0.04739
Hour 3		0.15596	0.0055
Hour 4		0.20804	0.00023
Hour 5		0.1985	0.00044
Hour 6		0.14471	0.01036
Hour 7		0.05316	0.35103
Hour 8		− 0.05456	0.34973
Hour 9		− 0.24203	0.00001
Hour 10		− 0.36265	< 0.00001
Hour 11		− 0.26118	< 0.00002
Hour 12		− 0.11427	0.0652
Hour 13		0.08329	0.18359
Hour 14		0.22539	< 0.00003
Hour 15		0.2807	< 0.00001
Hour 16		0.24684	0.00004
Hour 17		0.21301	0.00035
Hour 18		0.10169	0.08383
Hour 19		0.03344	0.56421
Hour 20		− 0.06257	0.27327
Hour 21		− 0.13673	0.01549
Hour 22		− 0.12945	0.02076
Hour 23		− 0.06749	0.22592

The overall dataset is split into 90:10 train-test datasets. The split was made to select samples proportionately (90% for training and 10% for testing) from each of the loggers^47^. After model development, the model was evaluated using the 10% withheld test dataset.

#### Mosquito population dynamics model

**Figure 7:**
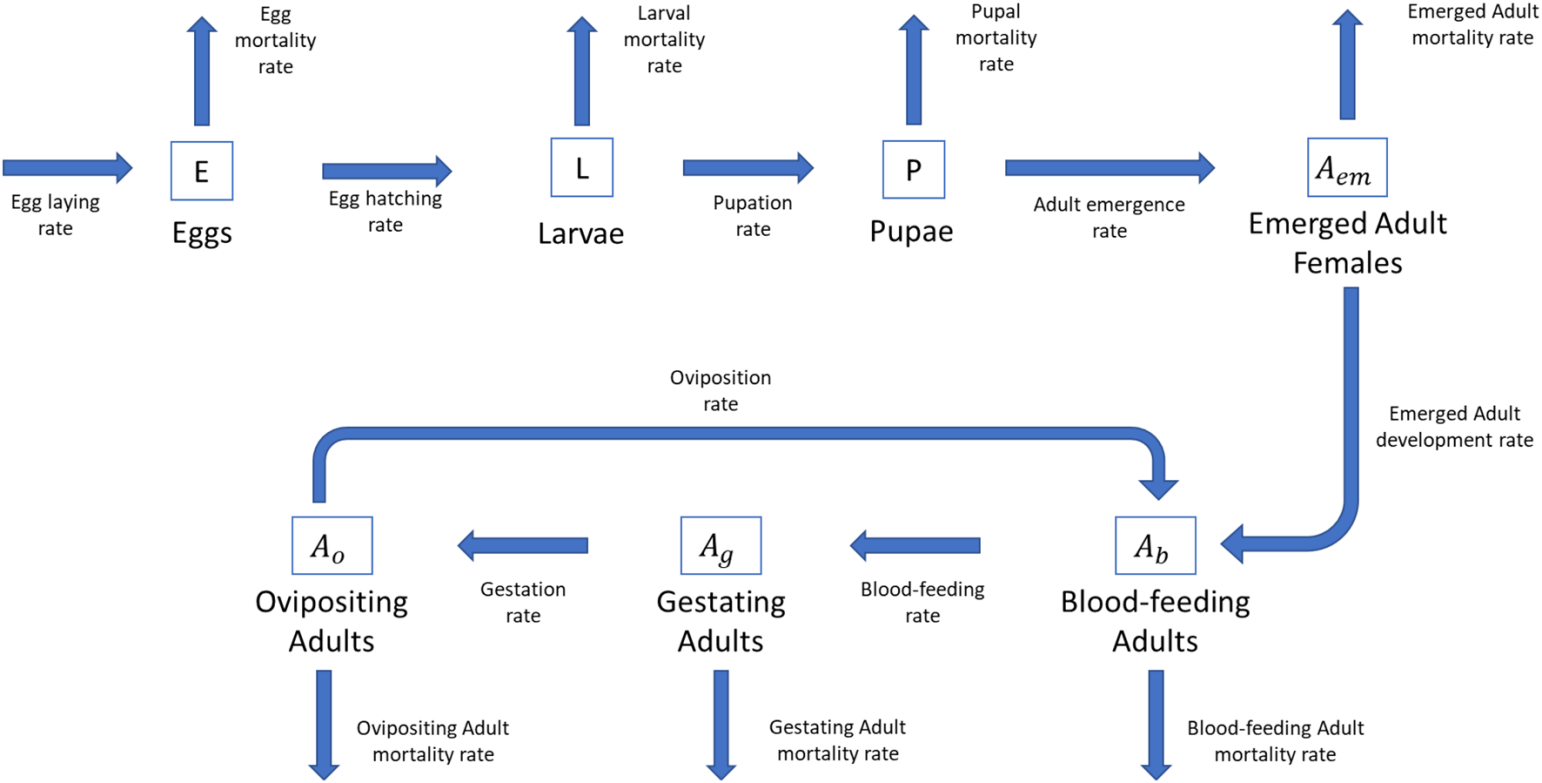
Population dynamics model.

A system dynamics model was developed to analyze the *Aedes albopictus* population dynamics under ambient and microclimatic conditions. The population model was developed to highlight the need for using accurate microclimatic temperature estimates. Mosquitoes have four life stages: eggs, larvae, pupae, and adults. The adult life stage can be divided into emerging, blood-feeding, gestating, and ovipositing physiological stages^48^. Overwintering or diapause is not explicitly included in the model, but the effect of temperature on development rates are included. The development from one stage to the next and the mortality at each stage were temperature-driven according to the environmental conditions (Fig. 7).

Ambient weather conditions at Hobby International Airport in Houston from January 1, 2013 to March 25, 2019 was used in the development of population dynamics model under ambient conditions due to proximity of the microclimatic logger used in the population model development. The model was run using the dataset of temperatures from January 1, 2013 to June 15, 2018 to initialize a stable starting population of each life stage. The remainder of the dataset was used as the ambient temperature data for simulating the population dynamics. Microclimate data of a storm drain in Houston from June 15, 2018 to March 25, 2019 was collected and used to run the alternate microclimate-based mosquito population model.

An hourly timestep based model was constructed and executed for the analysis of temperature influence on population dynamics. A series of differential equations were used to model the rate of changes in each of the life stages (Eq. 1). Parameters for the model are provided in Tables 2 and 3.

1 $$   \begin{aligned}   \frac{{dE}}{{dt}} &  = \beta A_{o}  - \left( {f_{E}  + \mu _{E} } \right)E \\    \frac{{dL}}{{dt}} &  = f_{E} E - \mu _{L} \left( {L + \frac{L}{{k_{L} }}} \right) - f_{L} L \\    \frac{{dP}}{{dt}} &  = f_{L} L - \left( {f_{P}  + \mu _{P} } \right)P \\    \frac{{dA_{{em}} }}{{dt}} &  = Pf_{P} \sigma e^{{\mu _{{A_{{em}} }} \left( {1 + \frac{P}{{k_{P} }}} \right)}}  - \left( {\mu _{{A_{{em}} }}  + \mu _{r} } \right)A_{{em}}  + f_{{A_{{em}} }} A_{{em}}  \\    \frac{{dA_{b} }}{{dt}} &  = f_{{A_{{em}} }} A_{{em}}  + f_{{A_{o} }} A_{o}  - \left( {\mu _{A}  + \mu _{r} } \right)A_{b}  - f_{{A_{b} }} A_{b}  \\    \frac{{dA_{g} }}{{dt}} &  = f_{{A_{b} }} A_{b}  - \mu _{A} A_{g}  - f_{{A_{g} }} A_{g}  \\    \frac{{dA_{o} }}{{dt}} &  = f_{{A_{g} }} A_{g}  - (\mu _{A}  + \mu _{r} )A_{o}  - f_{{A_{o} }} A_{o}  \\  \end{aligned}  $$

**Table 2:** Temperature-dependent variables.

Parameter	Definition	References
$f_{E}$	Egg hatching rate	^30,49,50^
$f_{L}$	Larval development rate	^50^
$f_{P}$	Pupal development rate	^50^
$\mu_{L}$	Larval mortality rate	^2^
$\mu_{P}$	Pupal mortality rate	^2^
β	Oviposition rate by each female	^50,51^
$f_{{A_{g} }}$	Gestating adult development rate	^2,30,49^
$\mu_{A}$	Adult mortality rate	^50^

**Table 3:** Constant parameters.

Parameter	Definition	Value	References
$\mu_{E}$	Egg mortality rate	0.05	^1,2^
$\mu_{{A_{em} }}$	Emerging adult mortality rate	0.1	^2^
$\mu_{r}$	Adult mortality related to risky behavior	0.08	^2,52^
$f_{{A_{em} }}$	Emerging adult development rate	0.4	^2^
$f_{{A_{b} }}$	Blood-feeding adult development rate	0.2	^2,52^
$f_{{A_{o} }}$	Ovipositing adult development rate	0.2	^2,30,52^
$k_{L}$	Larval carrying capacity	250,000	^1,2^
$k_{P}$	Pupal carrying capacity	250,000	^1,2^
σ	Percentage of females at emergence stage	0.5	^30^
$TDD_{{A_{g} }}$	Temperature development days required for gestation	77	^4^
$T_{{A_{g} }}$	Minimum temperature (°C) required for gestation	10	^4^

A 7-day moving average of the temperature data was used to run each of the models. We assume that breeding sites in storm drains are likely to be continuously supplied with nutrients due to run-off from irrigation systems. As a result, we assume constant carrying capacities for both larvae and pupae that are not impacted by precipitation. Additionally, adult blood-feeding mosquitoes were assumed to be inactive at temperatures below 9.5 °C^52^ and aquatic development stopped below 10 °C and above 40 °C^30^.

$$ {\text{Egg}}\;{\text{hatching}}\;{\text{rate:}}\; f_{E} \left( T \right) = 0.5070*{\text{exp}}\left[ { - \left( {\frac{T - 30.85}{{12.82}}} \right)^{2} } \right] $$

$$ {\text{Larval}}\;{\text{development}}\;{\text{rate:}}\;f_{L} \left( T \right) = 0.1727*{\text{exp}}\left[ { - \left( {\frac{T - 28.40}{{10.20}}} \right)^{2} } \right] $$

$$ {\text{Pupal}}\;{\text{development}}\;{\text{rate:}}\; f_{P} \left( T \right) = 0.6020*{\text{exp}}\left[ { - \left( {\frac{T - 34.29}{{15.07}}} \right)^{2} } \right] $$

$$ {\text{Larval}}\;{\text{mortality}}\;{\text{rate:}}\;\mu_{L} \left( T \right) = min\left\{ {e^{{ - \left( \frac{T}{2} \right)}} + 0.08, 1} \right\} $$

$$ {\text{Pupal}}\;{\text{mortality}}\;{\text{rate:}}\; \mu_{P} \left( T \right) = min\left\{ {e^{{ - \left( \frac{T}{2} \right)}} + 0.03, 1} \right\} $$

$$ {\text{Gestation}}\;{\text{rate:}}\; f_{{A_{g} }} \left( T \right) = \frac{{\left( {T - T_{{A_{g} }} } \right)}}{{TDD_{{A_{g} }} }}\quad {\text{if}}\;T > T_{{A_{g} }} ,\quad 0\;{\text{otherwise}} $$

$$ {\text{Eggs}}\;{\text{produced:}}\; B\left( T \right) = max\left\{ { - 15.837 + 1.289T - 0.0163T^{2} , 0} \right\} $$

$$ {\text{Adult}}\;{\text{mortality}}\;{\text{rate:}}\; \mu_{A} \left( T \right) = min\left\{ {\frac{1}{{\left| { - 0.1921*T^{2} + 8.147*T - 22.98} \right|}}, 1} \right\} $$

## Results and discussion

### Microclimate prediction model

The final model with significant features and corresponding p-values are shown in Table 1. This model resulted an R^2^ value of 94.78%. Significant variables include season (month), hour of the day, depth of the storm drain, ambient temperature, precipitation, relative humidity, atmospheric pressure, diurnal temperature range in the last 24 h, moving average of ambient temperature in the last 7 h, squares of the ambient temperature and diurnal temperature range. The overall root mean-square error (RMSE) when the model is evaluated on the independent test dataset is 1.57 °C. A logger-wise characterization of model performance can be found in Table 4.

**Table 4:** Prediction performance for different loggers.

Logger ID	Zip code	RMSE
U43	77,336	1.83
U40	77,346	1.65
U84	77,379	1.43
U76	77,069	1.53
U39	77,070	1.58
U69	77,520	1.16
U41	77,008	1.59
U59	77,521	2.15
**Overall**		**1.57**

**Figure 8:**
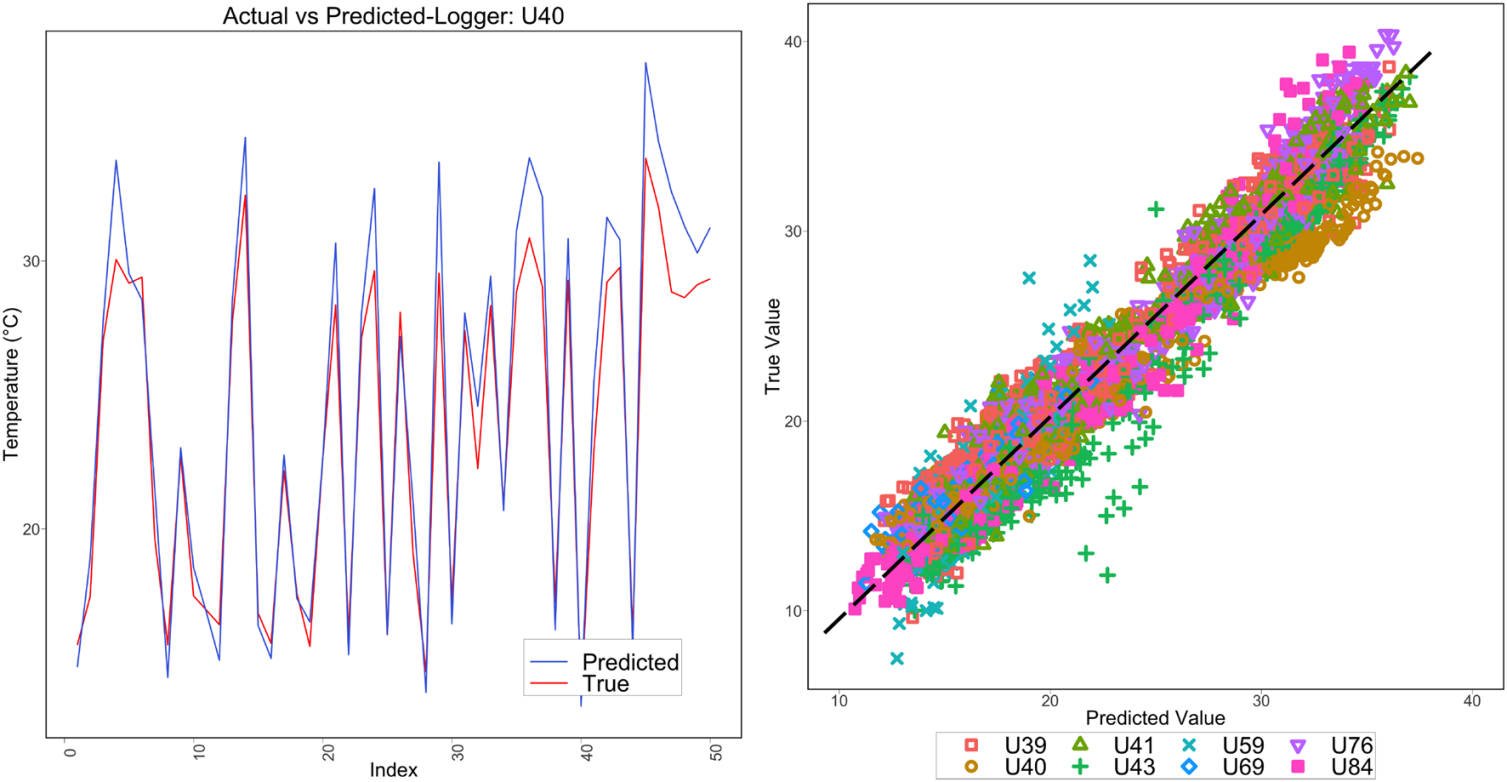
(Left) time sequenced actual versus predicted values for U40; (Right) actual vs predicted values of all the loggers.

The comparison of time-indexed actual and predicted values for a sample logger (U40) is shown in Fig. 8(Left). Comparison of actual and predicted values for all the loggers is summarized in Fig. 8(Right).

**Figure 9:**
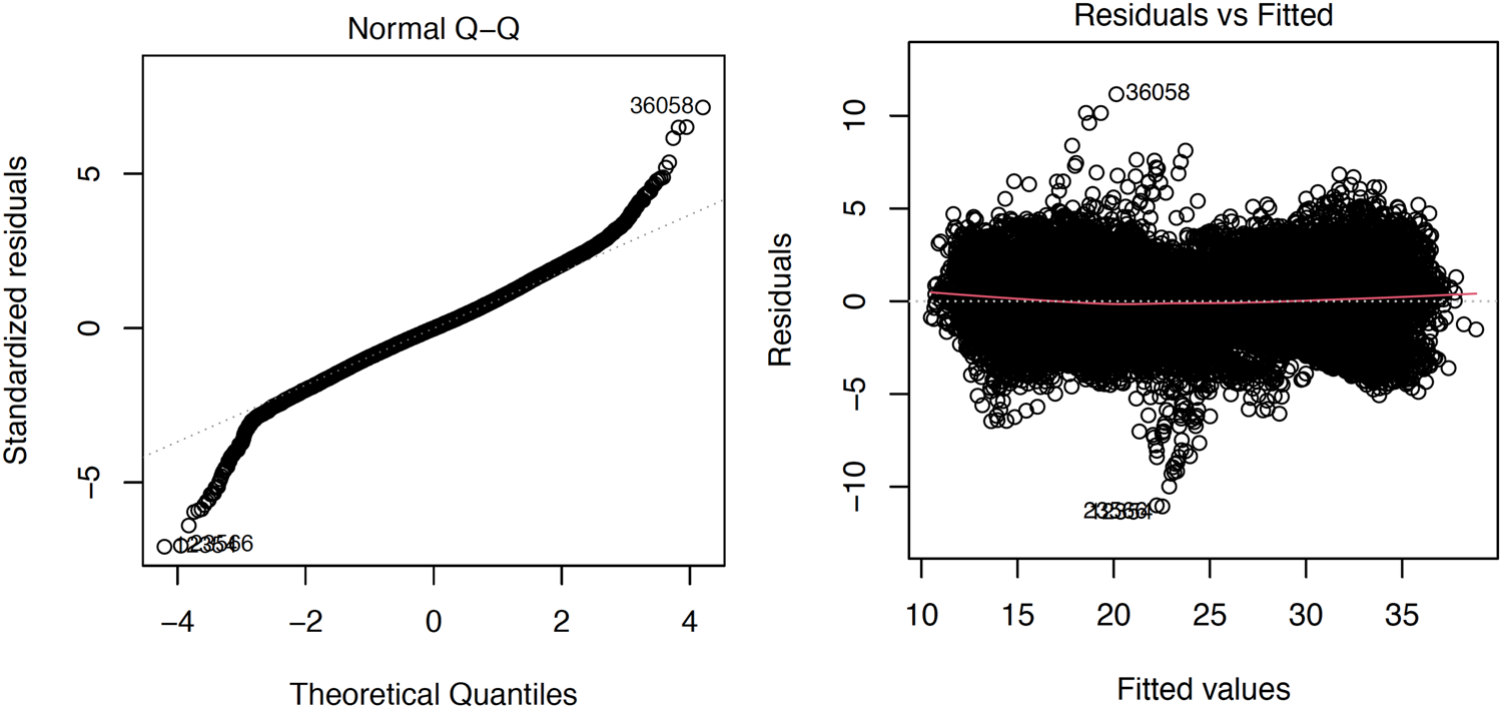
Linear regression diagnostic plots: (Left) QQ-plot (Right) residual plots.

The high R^2^ of the regression model and low RMSE on the test data indicates the validity of the developed microclimate prediction model. The regression diagnostic plots also conform the fit of the developed regression model (Fig. 9).

### Mosquito population model

**Figure 10:**
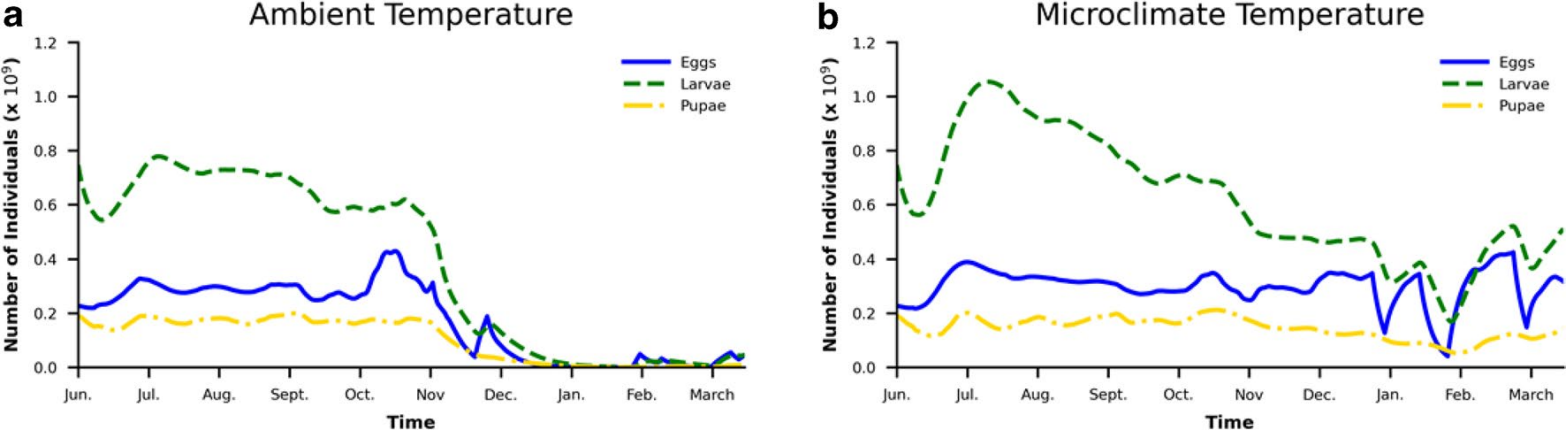
Eggs and Juvenile population under (**a**) ambient and (**b**) microclimatic conditions.

**Figure 11:**
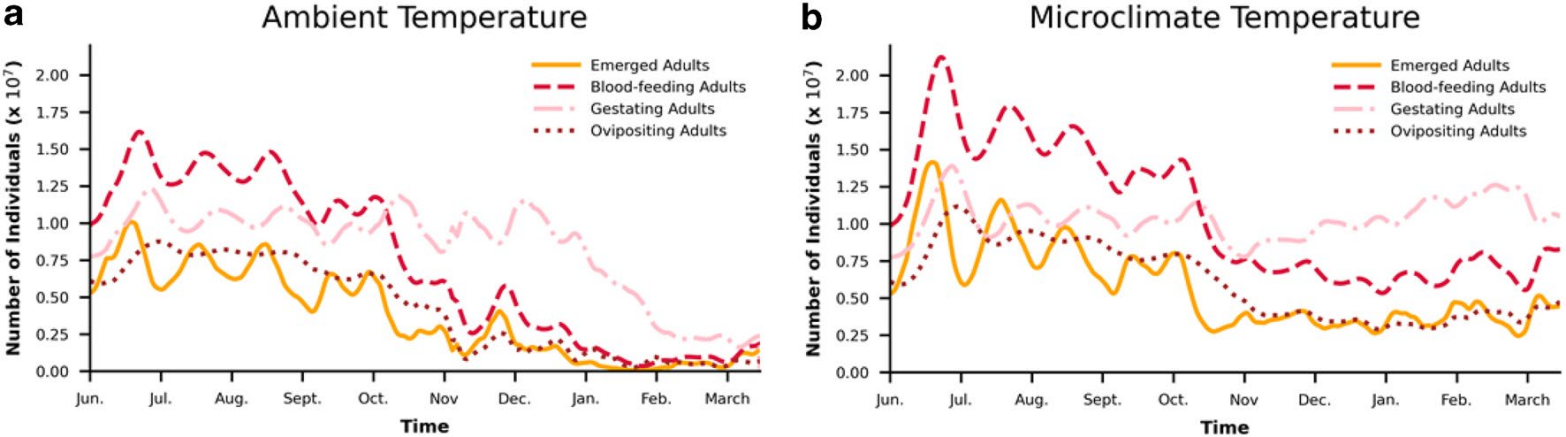
Adult mosquito population under (**a**) ambient and (**b**) microclimatic conditions.

The juvenile and adult population dynamics under ambient and microclimatic conditions are summarized in Figs. 10 and 11.

Under ambient condition modeling, the egg and juvenile population is almost reduced to zero and adult mosquito population is reduced to 23% during the winter time (Left panes in Figs. 10, 11). However, when microclimatic conditions are used, the insulated conditions in the storm drain results in the survival of 84% of juvenile and eggs and 96% of adults during the winter time (Right panes in Figs. 10, 11). It can be inferred that storm drains which are potential developmental and resting sites for mosquitoes^53^provide enough insulation from ambient weather conditions to facilitate juveniles and adults surviving the cold conditions without requiring dormancy. The results reinforce the necessity to use accurate micoclimate estimates for reliable analysis of mosquito population dynamics.

## Conclusions

Accurate analysis of mosquito population dynamics requires information on microclimatic conditions at breeding and resting locations. We explore the concept and utility of microclimatic prediction model that can be used to infer microclimatic conditions at storm drains, a potential breeding and nesting location, based on ambient conditions and contextual information, thereby eliminating the need for implementing complex sensor data collection and processing systems. Microclimatic temperatures in storm drains might provide enough insulation from ambient weather conditions to facilitate juveniles and adults avoid overwintering. The prediction model developed has high performance on training (R^2^) and test (RMSE) datasets validating the feasibility of the approach. The results are based on multiple loggers placed in storm drains in Houston. Collection of data from different geographical (tropical and sub-tropical) locations and vegetation, and development of predictive models for other microclimatic conditions such as humidity and light intensity will help generalize the model to these locations and conditions. We also present system dynamics-based *Aedes albopictus* mosquito population dynamics models to characterize the effect of the temperature on mosquito populations. Comparison of the model results based on ambient and microclimatic conditions indicate the necessity of using accurate temperature estimates in population dynamics analysis and thus the utility of microclimatic prediction model presented in this study.

## References

[1] Erickson RA, Presley SM, Allen LJ, Long KR, Cox SB (2010). A stage-structured, *Aedes albopictus* population model. Ecol. Model..

[2] Tran A (2013). A rainfall-and temperature-driven abundance model for *Aedes albopictus* populations. Int. J. Environ. Res. Public Health.

[3] Brady OJ (2012). Refining the global spatial limits of dengue virus transmission by evidence-based consensus. PLoS Negl. Trop. Dis..

[4] Mordecai EA (2017). Detecting the impact of temperature on transmission of Zika, dengue, and chikungunya using mechanistic models. PLoS Negl. Trop. Dis..

[5] Bellone R, Failloux A-B (2020). Temperature in shaping mosquito-borne viruses transmission. Front. Microbiol..

[6] Wimberly MC (2020). Land cover affects microclimate and temperature suitability for arbovirus transmission in an urban landscape. PLoS Negl. Trop. Dis..

[7] Erraguntla, M., Ramachandran, S., Wu, C.-N. & Mayer, R. J. Avian influenza datamining using environment, epidemiology, and etiology surveillance and analysis toolkit (E3SAT). In *2010 43rd Hawaii International Conference on System Sciences.* 1–7 (IEEE).

[8] Ramachandran, S., Erraguntla, M., Mayer, R. & Benjamin, P. Data mining in military health systems-clinical and administrative applications. In *2007 IEEE International Conference on Automation Science and Engineering.* 158–163 (IEEE).

[9] Hess A, Davis J, Wimberly M (2018). Identifying environmental risk factors and mapping the distribution of West Nile virus in an endemic region of North America. GeoHealth.

[10] Johnson TL (2017). Modeling the environmental suitability for Aedes (Stegomyia) aegypti and Aedes (Stegomyia) albopictus (Diptera: Culicidae) in the contiguous United States. J. Med. Entomol..

[11] Liu-Helmersson J, Stenlund H, Wilder-Smith A, Rocklöv J (2014). Vectorial capacity of *Aedes aegypti*: Effects of temperature and implications for global dengue epidemic potential. PloS One.

[12] Murdock CC, Evans MV, McClanahan TD, Miazgowicz KL, Tesla B (2017). Fine-scale variation in microclimate across an urban landscape shapes variation in mosquito population dynamics and the potential of *Aedes albopictus* to transmit arboviral disease. PLoS Negl. Trop. Dis..

[13] Ogden NH, Milka R, Caminade C, Gachon P (2014). Recent and projected future climatic suitability of North America for the Asian tiger mosquito *Aedes albopictus*. Parasit. Vectors.

[14] Proestos Y (2015). Present and future projections of habitat suitability of the Asian tiger mosquito, a vector of viral pathogens, from global climate simulation. Philos. Trans. R. Soc. B Biol. Sci..

[15] Rochlin I, Ninivaggi DV, Hutchinson ML, Farajollahi A (2013). Climate change and range expansion of the Asian tiger mosquito (*Aedes albopictus*) in Northeastern USA: Implications for public health practitioners. PloS One.

[16] Siraj A (2014). Altitudinal changes in malaria incidence in highlands of Ethiopia and Colombia. Science.

[17] Erraguntla, M. *et al.* Data integration and predictive analysis system for disease prophylaxisin. In *Proceedings of the 50th Hawaii International Conference on System Sciences* (2017).

[18] Freeze, J., Erraguntla, M. & Verma, A. Data integration and predictive analysis system for disease prophylaxis: Incorporating dengue fever forecastsin. In *Proceedings of the 51st Hawaii International Conference on System Sciences* (2017).

[19] McGowan CJ (2019). Collaborative efforts to forecast seasonal influenza in the United States, 2015–2016. Sci. Rep..

[20] Johnson LR (2015). Understanding uncertainty in temperature effects on vector-borne disease: A Bayesian approach. Ecology.

[21] Lowe R (2018). Nonlinear and delayed impacts of climate on dengue risk in Barbados: A modelling study. PLoS Med..

[22] Ryan SJ, Carlson CJ, Mordecai EA, Johnson LR (2019). Global expansion and redistribution of Aedes-borne virus transmission risk with climate change. PLoS Negl. Trop. Dis..

[23] Tesla B (2018). Temperature drives Zika virus transmission: Evidence from empirical and mathematical models. Proc. R. Soc. B Biol. Sci..

[24] Johansson MA, Powers AM, Pesik N, Cohen NJ, Staples JE (2014). Nowcasting the spread of chikungunya virus in the Americas. PloS One.

[25] Ruiz-Moreno D, Vargas IS, Olson KE, Harrington LC (2012). Modeling dynamic introduction of Chikungunya virus in the United States. PLoS Negl. Trop. Dis..

[26] Zhang Q (2017). Spread of Zika virus in the Americas. Proc. Natl. Acad. Sci..

[27] Zapletal J, Erraguntla M, Adelman ZN, Myles KM, Lawley MA (2018). Impacts of diurnal temperature and larval density on aquatic development of *Aedes aegypti*. PLoS One.

[28] Zapletal J (2019). Predicting aquatic development and mortality rates of *Aedes aegypti*. PloS One.

[29] Zapletal J (2021). Making gene drive biodegradable. Philos. Trans. R. Soc. B.

[30] Delatte H, Gimonneau G, Triboire A, Fontenille D (2009). Influence of temperature on immature development, survival, longevity, fecundity, and gonotrophic cycles of *Aedes albopictus*, vector of chikungunya and dengue in the Indian Ocean. J. Med. Entomol..

[31] Couret J, Dotson E, Benedict MQ (2014). Temperature, larval diet, and density effects on development rate and survival of *Aedes aegypti* (Diptera: Culicidae). PloS One.

[32] Eisen L (2014). The impact of temperature on the bionomics of Aedes (Stegomyia) aegypti, with special reference to the cool geographic range margins. J. Med. Entomol..

[33] Focks DA, Haile D, Daniels E, Mount GA (1993). Dynamic life table model for *Aedes aegypti* (Diptera: Culicidae): Analysis of the literature and model development. J. Med. Entomol..

[34] Kamimura K (2002). Effect of temperature on the development of *Aedes aegypti* and *Aedes albopictus*. Med. Entomol. Zool..

[35] Rueda L, Patel K, Axtell R, Stinner R (1990). Temperature-dependent development and survival rates of *Culex quinquefasciatus* and *Aedes aegypti* (Diptera: Culicidae). J. Med. Entomol..

[36] Tun-Lin W, Burkot T, Kay B (2000). Effects of temperature and larval diet on development rates and survival of the dengue vector *Aedes aegypti* in north Queensland, Australia. Med. Vet. Entomol..

[37] Benedict MQ, Levine RS, Hawley WA, Lounibos LP (2007). Spread of the tiger: Global risk of invasion by the mosquito *Aedes albopictus*. Vector Borne Zoonotic Dis..

[38] Evans MV (2019). Microclimate and larval habitat density predict adult *Aedes albopictus* abundance in urban areas. Am. J. Trop. Med. Hyg..

[39] Kearney M, Porter WP, Williams C, Ritchie S, Hoffmann AA (2009). Integrating biophysical models and evolutionary theory to predict climatic impacts on species’ ranges: The dengue mosquito *Aedes aegypti* in Australia. Funct. Ecol..

[40] Kraemer MU (2015). The global distribution of the arbovirus vectors *Aedes aegypti* and *Ae. albopictus*. eLife.

[41] Medley KA (2010). Niche shifts during the global invasion of the Asian tiger mosquito, *Aedes albopictus* Skuse (Culicidae), revealed by reciprocal distribution models. Glob. Ecol. Biogeogr..

[42] Zhu Y (2020). Classifying major depressive disorder using fNIRS during motor rehabilitation. IEEE Trans. Neural Syst. Rehabil. Eng..

[43] Sanders JD, Talley JL, Frazier AE, Noden BH (2020). Landscape and anthropogenic factors associated with adult *Aedes aegypti* and *Aedes albopictus* in small cities in the southern Great Plains. Insects.

[44] Crepeau TN (2013). Effects of Biogents Sentinel trap field placement on capture rates of adult Asian tiger mosquitoes, *Aedes albopictus*. PloS One.

[45] Evans MV (2019). Microclimate and larval habitat density predict adult *Aedes albopictus* abundance in urban areas. Am. J. Trop. Med. Hyg..

[46] Administration, N. O. a. A. (National Oceanic and Atmospheric Administration, National Oceanic and Atmospheric Administration).

[47] Dave, D. *et al.* Feature-based machine learning model for real-time hypoglycemia prediction. *J. Diabetes Sci. Technol.* 1932296820922622 (2020). 10.1177/1932296820922622 PMC8258517 32476492

[48] Clements A (1999). The Biology of Mosquitoes, Sensory Reception and Behavior.

[49] Craig MH, Snow R, le Sueur D (1999). A climate-based distribution model of malaria transmission in sub-Saharan Africa. Parasitol. Today.

[50] Jia P (2016). A climate-driven mechanistic population model of *Aedes albopictus* with diapause. Parasit. Vectors.

[51] Yang H, Macoris M, Galvani K, Andrighetti M, Wanderley D (2009). Assessing the effects of temperature on the population of Aedes aegypti, the vector of dengue. Epidemiol. Infect..

[52] Luciano T, Severini IF, Di Luca IM, Bella IA, RryP Roberto R (2003). Seasonal patterns of oviposition and egg hatching rate of *Aedes albopictus* in Rome. J. Am. Mosq. Control Assoc..

[53] Su T, Webb JP, Meyer RP, Mulla MS (2003). Spatial and temporal distribution of mosquitoes in underground storm drain systems in Orange County, California. J. Vector Ecol..

